# Set3 Is Required for Asexual Development, Aflatoxin Biosynthesis, and Fungal Virulence in *Aspergillus flavus*

**DOI:** 10.3389/fmicb.2019.00530

**Published:** 2019-03-29

**Authors:** Huahui Lan, Lianghuan Wu, Kun Fan, Ruilin Sun, Guang Yang, Feng Zhang, Kunlong Yang, Xiaolu Lin, Yanhong Chen, Jun Tian, Shihua Wang

**Affiliations:** ^1^ Key Laboratory of Pathogenic Fungi and Mycotoxins of Fujian Province, Key Laboratory of Biopesticide and Chemical Biology of Education Ministry, and School of Life Sciences, Fujian Agriculture and Forestry University, Fuzhou, China; ^2^ College of Life Science, Jiangsu Normal University, Xuzhou, China; ^3^ Longyan City Corporation of Fujian Tobacco Corporation, Longyan, China

**Keywords:** Set3, regulate, reproduction, aflatoxin biosynthesis, *Aspergillus flavus*

## Abstract

*Aspergillus flavus* is an opportunistic pathogenic fungus for both plant and animal that produces carcinogenic toxins termed aflatoxins (AFs). To identify possible genetic targets to reduce AF contamination, in this study, we have characterized a novel *A. flavus* Set3, and it shares sequence homology with the yeast protein Set3. The *set3* deletion mutants present no difference in growth rate but alterations in asexual development and secondary metabolite production when compared to the *A. flavus* wild type. Specifically, deletion of *set3* gene decreases conidiophore formation and conidial production through downregulating expression of *brlA* and *abaA* genes. In addition, normal levels of *set3* are required for sclerotial development and expression of sclerotia-related genes *nsdC* and *sclR*. Further analyses demonstrated that Set3 negatively regulates AF production as well as the concomitant expression of genes in the AF gene cluster. Importantly, our results also display that *A. flavus* Set3 is involved in crop kernel colonization. Taking together, these results reveal that a novel Set3 plays crucial roles in morphological development, secondary metabolism, and fungal virulence in *A. flavus*.

## Introduction

As both plant and animal opportunistic pathogenic fungus, *Aspergillus flavus* is responsible for serious health and economic impacts worldwide by producing carcinogenic mycotoxins termed aflatoxins (AFs). Many agriculturally important oilseed crops, such as peanuts, maize, and tree nuts, can be contaminated by *A. flavus* and AFs (Amaike and Keller, 2011). AFs are also responsible for numerous health problems, including acute aflatoxicosis, immunosuppression, liver cancer, and even death in many animal species and human. These diseases are highly linked to the consumption of large amounts of AFs due to ingestion of contaminated crops (Hedayati et al., 2007; Klich, 2007). Economically, AF contamination leads to substantial monetary losses yearly, due in large part to rejection or reduced value of contaminated crops as well as costs associated with monitoring and detection in developed countries (Wu et al., 2014). AFs and other mycotoxins are estimated to contaminate one quarter of the world’s crops. Specifically, the health risks are a major concern in developing countries because of the lack of strict regulations or monitoring AF levels in commodities prior to consumption (Groopman et al., 2008).

Nowadays, approaches such as chemical or physical methods are insufficient to control *A. flavus* colonization and AF contamination, since *A. flavus* caused extensive infestations by generating asexual spores called conidia (Adams et al., 1998; Hedayati et al., 2007; Amaike and Keller, 2011). New strategies, such as those depending on genetic approaches, could contribute to the development of new methodologies to decrease dissemination and survival of this organism, as well as AF biosynthesis. Therefore, it is quite important to explore genetic regulatory pathways that control *A. flavus* morphogenesis and AF biosynthesis. Previous studies have revealed that AF biosynthesis is controlled by regulatory cluster pathways (Payne and Brown, 1998; Yu et al., 2004), and increasing literatures showed that AFs are regulated not only by cluster genes (Amare and Keller, 2014; Nie et al., 2018) but also by other signal pathways (Roze et al., 2004), transcriptional regulators (Affeldt et al., 2014; Cary et al., 2017; Chang et al., 2017), and epigenetic regulators (Lan et al., 2016; Zhi et al., 2017; Pfannenstiel et al., 2018).

Set3 is a signature of chromatin-associated protein, which was first characterized in yeast by its feature of containing plant homeodomain (PHD) finger and Su(var)3-9, Enhancer-of-zeste, Trithorax (SET) domains (Pijnappel et al., 2001). Nowadays, Set3 protein had been identified in various eukaryotic cells, and these proteins encompass several roles, such as histone methyltransferase activity, and protein-protein interactions with other factors involved in chromatin regulation. Current data demonstrated that Set3 participates in multiple cellular functions, including meiosis-specific repression of sporulation (Pijnappel et al., 2001), promotion of Ty1 retrotransposon integration at tRNA genes (Mou et al., 2006), signaling secretory stress upon the PKC cell integrity pathway (Cohen et al., 2008), the white-opaque transition and pathogenicity in *Candida albicans* (Hnisz et al., 2010), as well as the environmental stress response (Torres-Machorro et al., 2015; Yu et al., 2016). In budding yeast, Set3, Hos2, Sif2, and Snt1 form the functional core of a histone deacetylase complex named Set3/Hos2 complex (Set3C) (Pijnappel et al., 2001). Recently, Set3C is found to play both repressive and activating roles in transcription, depending on the context of the region to which it is recruited (Kim and Buratowski, 2009). Set3C is predominantly recruited to the 5′ transcribed region of genes to reduce the histone acetylation level (Hnisz et al., 2012). A recent study also showed that Set3 can regulate transcription independent of Set3C (Yu et al., 2016).

Although the roles of Set3 in many organisms have been studied, the function of Set3 in *A. flavus* has not been characterized. Herein, by using gene knockout strategy, we identified a novel Set3 in *A. flavus*, encoding a putative SET and a PHD domain protein. Our results reveal that Set3 is involved in morphological development, secondary metabolism, and virulence of the agriculturally and medically important fungus *A. flavus*.

## Materials and Methods

### Strains and Growth Conditions

The uracil auxotrophic strain *A. flavus* PTSΔ*ku70*Δ*pyrG* (SRRC collection number 1709) (Chang et al., 2010) was used as recipient strain for gene knockout, and PTSΔ*ku70*Δ*pyrG:*: *AfpyrG* was used as wild-type strain (WT). For phenotype assays, all utilized strains were cultured on potato dextrose agar (PDA, BD Difco™, USA) media for growth assays at 37°C, on yeast extract sucrose (YES, 20 g/l yeast extract, 150 g/l sucrose, 1g/l MgSO_4_•7H_2_O) media at 29°C for aflatoxin analysis, and on sclerotia-inducing Wickerham media (WKM, 2 g/l yeast extract, 3 g/l peptone, 5 g/l cornsteep solids, 2 g/l dextrose, 30 g/l sucrose, 2 g/l NaNO_3_, 1 g/l K_2_HPO_4_•3H_2_O, 0.5 g/l MgSO_4_•7H_2_O, 0.2 g/l KCl, 0.1 g/l FeSO_4_•7H_2_O) (Lan et al., 2016) for sclerotia analysis. Each strain was cultured on three plates at least for technical replicates, and each experiment was repeated for three times.

### Phylogenetic Tree and Domain Analysis

Amino acid sequences of *Saccharomyces cerevisiae* Set3 (GenBank accession number: NP_012954.3) were used as a query, and basic local alignment search tool algorithm was used to download sequences of Set3 protein (*Aspergillus* spp. *Candida albicans*, *Fusarium graminearum*, *Magnaporthe oryzae*, *Neurospora crassa*, *Arabidopsis thaliana*, *Drosophila melanogaster*, *Danio rerio*, *Mus musculus*, *Homo sapiens*) from National Center for Biotechnology Information resources (NCBI, http://www.ncbi.nlm.nih.gov/). A neighbor-joining phylogenetic tree was constructed by the MEGA 6.0 software. The visualized Set3 domain was generated by DOG 2.0 software (downloaded from http://dog.biocuckoo.org/).

### Construction of Knockout and Complemented Mutant Strains

To construct s*et3* knockout mutant (Δs*et3*) strain, previous approach was used (Yang et al., 2016a) Primers utilized in this study were listed in Table 1. The entire gene deletion cassettes were amplified with specific primers. Overlap polymerase chain reaction (PCR) method was performed as described earlier (Szewczyk et al., 2006), and then, fusion PCR products were transformed into the PTSΔ*ku70*Δ*pyrG* protoplasts of *A. flavus*. For constructing *set3* complemented (Δ*set3-com*) strain, PCR products of native promoter and open reading frame for Set3, combined with plasmid pPTR1 (Takara, Japan) containing the marker gene *ptrA*, were re-introduced into the protoplasts of the gene deletion strains. Fungal transformants were preliminary analyzed by PCR and reverse transcription PCR (RT-PCR) and further verified by southern blot as reported earlier in our group (Yang et al., 2016a).

**Table 1:** Primers utilized in this study.

Primer	Sequence (5’-3’)
*set3*-AF *set3*-AR	CAAGAAGATGTCACCCAACC GGGTGAAGAGCATTGTTTGAGGCCAACCGAGCCTGCCTAC
*set3*-BF *set3*-BR	GCATCAGTGCCTCCTCTCAGACCTCCTGCCGGTGGTGAT CAAGGTGGTTCTCGCTCC
*pyrG-*F *pyrG-*R	GCCTCAAACAATGCTCTTCACCC GTCTGAGAGGAGGCACTGATGC
*set3*-NF *set3*-NR	CACGAGATGGGTTCCTGAT GAGATGGTTGCGGTTGAG
*set3*-OF *set3*-OR	CTCTTTACATCCATCGGTTTC GTGGGTGCCGTTTACTTG
P801 P1020	CAGGAGTTCTCGGGTTGTCG CAGAGTATGCGGCAAGTCA
*set3*-com-F *set3*-com-R	TTGGCACATACGCAACTA TGATACGCCGTCACAAA
mCherry-AF mCherry-AR	ACCGAAGAAAGAAGCGAGCCA CTCGCCCTTGCTCACCATGGAAAGCGAGGATAGCTGGGA
mCherry-*ptr*-F mCherry-*ptr*-R	ATGGTGAGCAAGGGCGAG CGAGGTGCCGTAAAGCACTAACTACTTGTACAGCTCGTCCAT
*ptrA*-F *ptrA*-R	CCGATTTCGGTCTATTGGT CGACACGGAAATGTTGAA
mCherry-BF mCherry-BR	CTGGATGGAGGCGGATAAAGTCTCCTGCCGGTGGTGAT CAAGGTGGTTCTCGCTCC
mCherry-NF mCherry-NR	CCACTGCTGCTCATAACTC CCTAAACACCATACATACCCT

Each row may not add up to 100% because some of the options were not selected, although this only represents fewer than 2% of the choices.

### Microscopic Examination of Set3-mCherry Subcellular Localization

*A. flavus* Set3-mCherry strains were prepared using a published method (Yang et al., 2016b), and the primers were listed in Table 1. To assess Set3-mCherry localization, fresh mycelia were analyzed using the Leica confocal SP8 microscope (Leica, Heidelberg, Germany). The nuclei of mycelia were observed after samples were stained with 1 μg/ml 4′,6-diamidino-2-phenylindole (DAPI, Sigma, USA).

### Analysis of Fungal Conidia and Sclerotia

Spores (10^6^ conidia/ml) from each strain were top-agar inoculated on PDA media for conidia assays and on WKM media for sclerotia analysis. For conidia analysis, cultures were incubated at 37°C in darkness for 5 days, and conidia were collected in triplicate from 10-mm cores that taken from equivalent zones of the fungal surface of PDA, and the collected samples were homogenized and diluted in 3 ml of 0.05% Tween-20 and counted by a hemocytometer (Qiujing, Shanghai, China). For sclerotia analysis, after 7 days grown on WKM media, each plate was sprayed with 75% ethanol to wash away the mycelia mat to allow the enumeration of the sclerotia. Sclerotia were collected and counted with the light microscope (Leica, Heidelberg, Germany). Each strain was assessed on five plates, and each experiment was repeated three times.

### Stress Response Assays

The WT, the ∆*set3-1*, the ∆*set3-2*, and the ∆*set3-com* strains were inoculated onto PDA agar with oxidative stress agent H_2_O_2_ (2.5 and 5 mM) and cell wall stress agent Congo red (CR, 200 and 500 μg/ml), at 37°C in darkness for 3 days, respectively. To analyze the role of Set3 in stress response of *A. flavus*, the relative inhibition rates were calculated, according to the formula listed in the brackets {(diameter of colony without inhibitor − diameter of colony with inhibitor)/diameter of colony without inhibitor}. The experiments were performed in three repetitions.

### Determination of Aflatoxin Production

To analyze aflatoxins (AFs), each strain was cultured in YES liquid media at 29°C for 3 days (180 r/min). Extracted AF samples were assessed by thin layer chromatography (TLC) and high performance liquid chromatography (HPLC) methods as previously described (Lan et al., 2016).

Briefly, 10^6^ conidia of the WT, the ∆*set3-1*, the ∆*set3-2,* and the ∆*set3-com* strains were inoculated in 50 ml YES liquid medium, and cultures were incubated at 29°C. After 72 h, the cultures were combined with 25 ml chloroform in 250 ml flask, which were shaken for 30 min. The mycelia were then collected, dried completely, and weighed. Next, the organic layer of each sample was taken to a new plate, completely dried, and resuspended in chloroform solvent (1 ml/mg of mycelia). Then, the extracts (10 ml/sample) were loaded onto silica TLC plates (Haiyang Chemical, Qingdao, China) and separated in developing solvent (chloroform: acetone = 9:1). The TLC plates were exposed to UV radiation (365 nm) and photographed using a Quantum ST5 imaging system (Vilber Lourmat Deutschl and GmbH, Eberhardzell, Germany).

For HPLC experiment, the aflatoxin extracts were dissolved in methanol, filtered (0.22 μm), and performed by a Mycotox™ column (Waters, Milford, USA) at 42°C. The column was equilibrated in running solvent (water: methanol: acetonitrile = 56: 22: 22), and 10 μl samples were injected, and isocratic runs were conducted for 15 min in 100% running solvent at a flow rate of 1.0 ml/min. Aflatoxins were analyzed using a fluorescent detector (Waters, Milford, USA) with excitation and emission wave lengths of 365 and 455 nm, respectively. Aflatoxin production for each strain was analyzed using three flasks, and each experiment was repeated three times.

### Crops Infection Experiments

Peanuts and maize seed colonization assays were performed using a published procedure (Lan et al., 2016). The peanut cotyledons and maize seeds infected with utilized strains were incubated at 28°C. After 5 days incubation, host seeds were harvested in 50 ml Falcon tubes and then vortexed for 2 min to release conidia into 20 ml sterile water supplemented with 0.05% Tween-80. The aflatoxin from the infected host seeds was extracted and analyzed as previously described (Lan et al., 2016).

### Quantitative Real-Time PCR Analysis

For qRT-PCR analysis, mycelia of all tested strains were collected from PDA, WKM, and YES cultures for total RNA isolation with TRIzol reagent (Biomarker Technologies, Beijing, China). qRT-PCR was performed with Piko real-time PCR system (Thermo Fisher Scientific, Finland) by using the qPCR SuperMix (TransGen Biotech, Beijing, China). All utilized qRT-PCR primers were listed in Table 2. The relative quantification of expression level for each gene was calculated following the 2^−ΔΔCt^ method, and the expression of *actin* was used as internal control. Each sample for qRT-PCR assays was conducted with technical triplicates, and the experiment was repeated three times.

**Table 2:** qRT-PCR Primers utilized in this study.

Primer	Sequence (5’-3’)
*brlA*/QF *brlA*/QR	GCCTCCAGCGTCAACCTTC TCTCTTCAAATGCTCTTGCCTC
*abaA*/QF *abaA*/QR	CACGGAAATCGCCAAAGAC TGCCGGAATTGCCAAAG
*nsdC*/QF *nsdC*/QR	GCCAGACTTGCCAATCAC CATCCACCTTGCCCTTTA
*sclR*/QF *sclR*/QR	CAATGAGCCTATGGGAGTGG ATCTTCGCCCGAGTGGTT
*nsdD*/QF *nsdD*/QF	GGACTTGCGGGTCGTGCTA AGAACGCTGGGTCTGGTGC
*aflR*/QF *aflR*/QR	AAAGCACCCTGTCTTCCCTAAC GAAGAGGTGGGTCAGTGTTTGTAG
*aflS*/QF *aflS*/QR	CGAGTCGCTCAGGCGCTCAA GCTCAGACTGACCGCCGCTC
*aflC*/QF *aflC*/QR	GTGGTGGTTGCCAATGCG CTGAAACAGTAGGACGGGAGC
*aflD*/QF *aflD*/QR	GTGGTGGTTGCCAATGCG CTGAAACAGTAGGACGGGAGC
*aflK*/QF *aflK*/QR	GAGCGACAGGAGTAACCGTAAG CCGATTCCAGACACCATTAGCA
*aflO*/QF *aflO*/QR	GATTGGGATGTGGTCATGCGATT GCCTGGGTCCGAAGAATGC
*aflP*/QF *aflP*/QR	ACGAAGCCACTGGTAGAGGAGATG GTGAATGACGGCAGGCAGGT
*aflQ*/QF *aflQ*/QR	GTCGCATATGCCCCGGTCGG GGCAACCAGTCGGGTTCCGG
*actin*/QF *actin*/QR	ACGGTGTCGTCACAAACTGG CGGTTGGACTTAGGGTTGATAG

### Statistical Analysis

All data were presented with the means ± SD (standard deviation). The significant differences (statistical significances) among groups were calculated with ANOVA and least significant difference (LSD) tests. The statistical analysis and significance were performed with the software GraphPad Prism5 (La Jolla, CA, USA), and the difference is regarded to be statistically significant when *p* < 0.05.

## Results

### Identification and Analysis of Set3 in *A. flavus*

**Figure 1:**
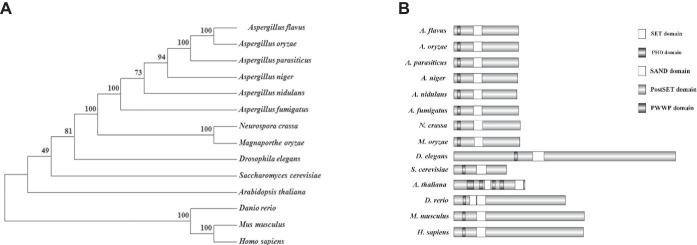
Characterization of Set3 protein of *A. flavus*. **(A)** Domains from Set3 proteins were characterized by SMART, and DOG2.0 software was used to visualize protein domains. **(B)** Phylogenetic relationship of Set3 from different species was analyzed.

There were no previous reports of Set3 in *Aspergillus* species, so the Set3 amino acid sequence from model fungus *Saccharomyces cerevisiae* (GenBank accession number: NP_012954.3) was used with a basic local alignment search tool algorithm, then a putative protein that contains a PHD finger and a SET domain protein was identified in *A. flavus* designated Set3 (AFLA_134050). *A. flavus* Set3 presents 24% identity and 52% similarity with *S. cerevisiae* Set3, while it showed 61% similarity with the model filamentous fungus *Aspergillus nidulans* (AN5891.2, a putative protein). Analysis of Set3 proteins indicated that all of those Set3 proteins share conserved structures consisting of SET and PHD domains among fungi, plants, and animals (Figure 1A). A phylogenetic tree of evolutionary relationship of these Set3 proteins was constructed, revealing that the Set3 protein is conserved among *Aspergillus* species (Figure 1B).

### Subcellular Localization of *A. flavus* Set3

**Figure 2:**
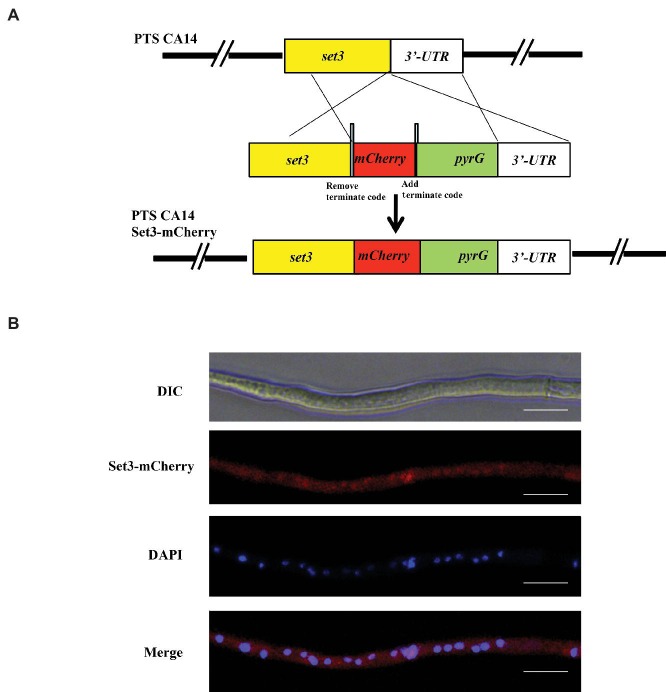
Subcellular localization of *A. flavus* Set3. **(A)** Construction strategy of Set3-mCherry strains. **(B)** Fluorescent image of Set3-mCherry during the hyphae growth period, and the nucleus was stained with DAPI. Bars = 20 μm.

For subcellular localization analysis, a Set3-mCherry fusion generated with its native promoter was constructed and transformed into *A. flavus* auxotrophic strain PTSΔ*ku70*Δ*pyrG*. The construction strategy was shown in Figure 2A, and the resulting transformed strains exhibited a similar phenotype with WT strain, suggesting that the mCherry-tag did not affect the function of Set3 of *A. flavus* (data not shown). The results in Figure 2B showed that the mCherry fluorescence was dispersed in whole cytoplasm. By staining with 4,6-diamidino-2-phenylindole (DAPI), we also found that *A. flavus* Set3 is localized not only in cytoplasm but also in nucleus (Figure 2B).

### *Set3* Does Not Affect Growth Rate, but Involves in Hyphal Development

**Figure 3:**
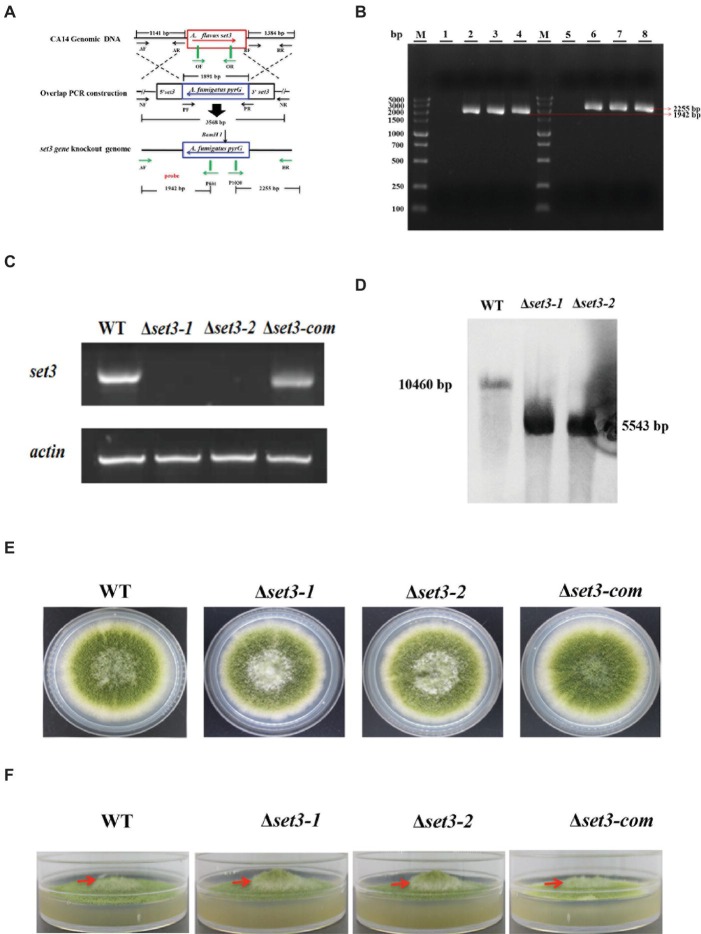
Construction of the *set3* deleted (Δ*set3-1* and Δ*set3-2*), complemented strains (Δset3-com), and growth analysis. **(A)** Construction strategy for Δ*set3* strain using homolog recombination. **(B)** The deleted and complemented strains were verified by PCR analysis with genomic DNA as template, lane 1~4: PCR examination on upstream of WT, Δ*set3-1*, Δ*set3-2,* and Δ*set3-com* strains, lane 5~8: PCR examination on downstream of WT, Δ*set3-1*, Δ*set3-2,* and Δ*set3-com* strains. (C) RT-PCR was used to confirm the transcript levels of *set3* gene in deleted and complemented strains. **(D)** Southern blot was conducted to confirm the deletion mutants. (E) Colony morphology of WT, Δ*set3,* and Δ*set3-com* strains, grown on PDA media at 37°C for 5 days. **(F)** The Δ*set3-1* and Δ*set3-2* strain showed fluffier phenotype when compared to WT and Δ*set3-com* strains.

To gain an insight into the function of Set3 in morphogenesis of *A. flavus*, we generated *set3* gene deletion mutants (Δ*set3-1* and Δ*set3-2*) and complementation strain (Δ*set3-com*), which are illustrated in Figure 3A. Transformants were confirmed by diagnostic PCR (Figure 3B). Expression levels of *set3* in WT, Δ*set3,* and Δ*set3-com* strains were analyzed by RT-PCR, and the results showed that *set3* gene transcript level was not expressed in those deletion strains, whereas *set3* was detected in both the WT and Δ*set3-com* strains (Figure 3C). The deletion strains were further verified by Southern blot (Figure 3D). In this study, we selected two deletion strains Δ*set3-1* and Δ*set3-2* for further analysis. In the morphological study, our results showed that colony growth was not significantly altered in Δ*set3* strains in comparison to the WT and Δ*set3-com* strains (Figure 3E). However, the Δ*set3-1* and Δ*set3-2* strains presented more fluffy phenotype when compared to WT and Δ*set3-com* strains (Figure 3F), suggesting that Set3 involves in hyphal growth in *A. flavus*.

### *Set3* Regulates Conidia Formation

**Figure 4:**
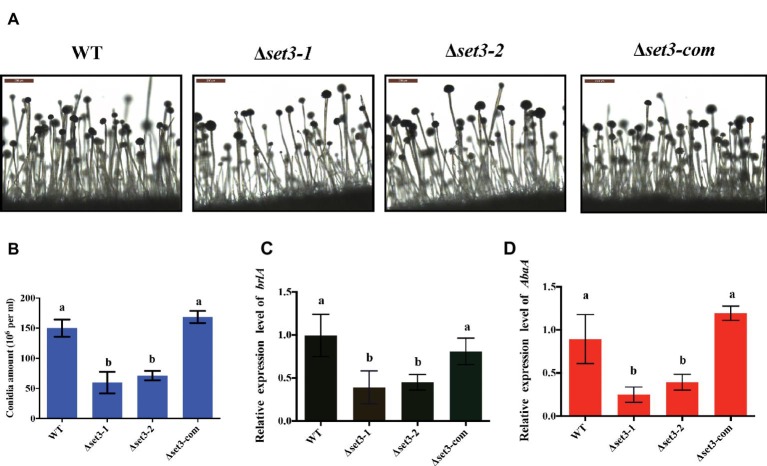
Deletion of *set3* caused defects of conidiation in *A. flavus*. (A) Conidiophores of WT, Δ*set3-1*, Δ*set3-2,* and Δ*set3-com* strains were observed by microscope after 12 h incubation, and bars = 200 μm. (B) Conidia production of WT, Δ*set3-1,* Δ*set3-2,* and Δ*set3-com* strains. (C) Transcript levels of conidia-related gene *brlA* among WT, Δ*set3-1,* Δ*set3-2,* and Δ*set3-com* strains. (D) Transcript levels of conidia-related gene *abaA* among WT, Δ*set3-1*, Δ*set3-2,* and Δ*set3-com* strains. Different letters represent p < 0.05.

In addition to fungal growth, Δ*set3-1* and Δ*set3-2* strains were found to decrease severely in conidiation when compared to WT strains (Figure 4B). For analysis of defect in conidiation, we further examined formation of conidiophores, and the result showed the Δ*set3-1* and Δ*set3-2* strains generate less normal conidiophores than WT strains (Figure 4A). Next, we checked the expression levels of genes *brlA* and *abaA*, which encode transcript factors related to conidiation. The results indicated that the transcript levels of both *brlA* (*p* < 0.05) and *abaA* (*p* < 0.05) were significantly reduced in the Δ*set3-1* and Δ*set3-2* strains, when compared to the WT andΔ*set3-com* strains (Figures 4C,D). All these results indicated that *set3* regulates conidia formation in *A. flavus.*

### *Set3* Positively Affects Sclerotia Production

**Figure 5:**
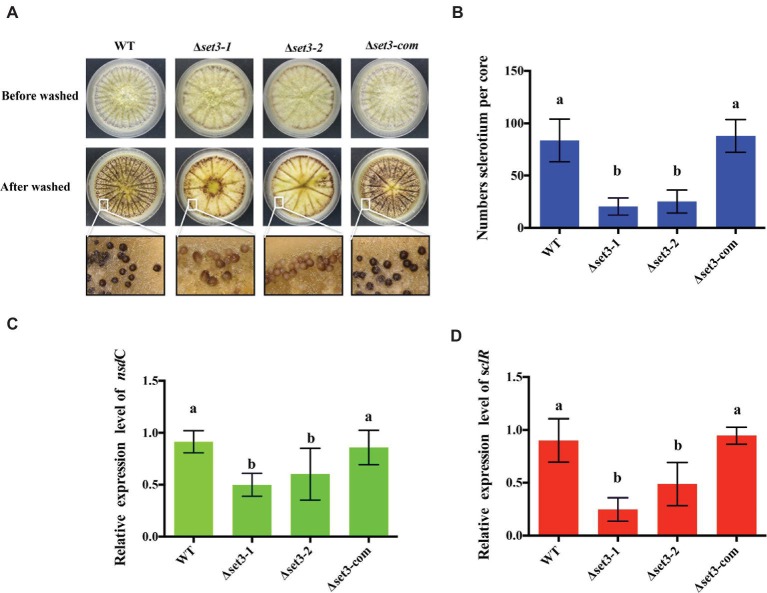
Deletion of *set3* caused defects of sclerotia production in *A. flavus*. **(A)** Phenotypic analyses of WT, Δ*set3-1,* Δ*set3-2,* and Δ*set3-com* strains grown on WKM media at 37°C for 7 days. **(B)** Sclerotia production of WT, Δ*set3-1,* Δ*set3-2,* and Δ*set3-com* strains. **(C,D)** Gene transcript level of sclerotia-related genes *nsdC* and *sclR* among WT, Δ*set3-1,* Δ*set3-2,* and Δ*set3-com* strains, respectively. Different letters represent *p* < 0.05.

*A. flavus* produces sclerotia to adapt unsuitable environment (Horn et al., 2009). To determine involvement of Set3 in sclerotia formation, all the strains were cultured on the sclerotia-inducing Wickerham media (WKM) at 37°C for 7 days. The results indicated that sclerotia production in the Δ*set3-1* and Δ*set3-2* strains was significantly decreased and less matured than that of the WT and complemented strains (*p* < 0.05) (Figures 5A,B). To confirm these findings, we performed qRT-PCR to check transcript levels of the sclerotia-related genes, *nsdC* and *sclR*. The results revealed that gene expression levels of *nsdC* (*p* < 0.05) and *sclR* (*p* < 0.05) were significantly lower in the Δ*set3-1* and Δ*set3-2* strains than WT and Δ*set3-com* strains (Figures 5C,D). These above results showed that *set3* plays an important role in sclerotia production in *A. flavus*.

### *Set3* Plays Important Roles in Responses to Oxidative and Cell Wall Stresses

**Figure 6:**
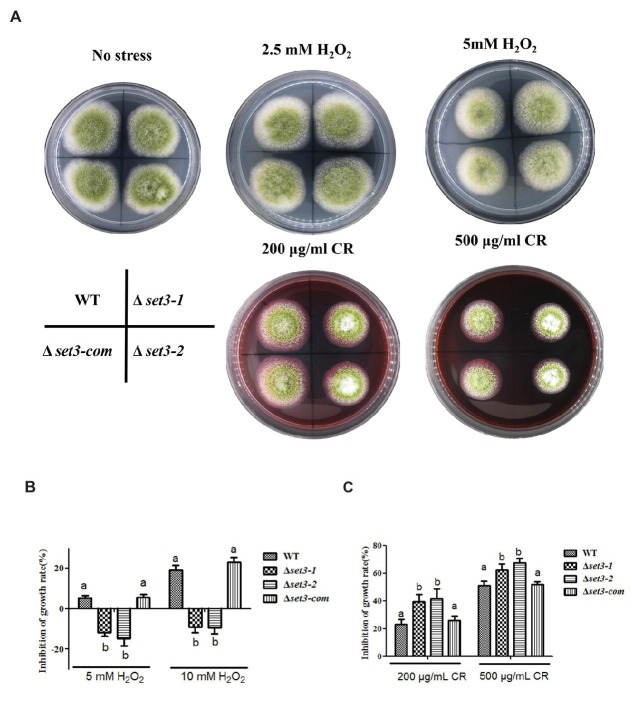
Deletion of *set3* affects oxidative and cell wall stress responses in *A. flavus*. **(A)** Colony morphology of WT, Δ*set3-1,* Δ*set3-2,* and Δ*set3-com* strains cultured on PDA media with oxidative stress agents (2.5 mM and 5 mM H_2_O_2_) and cell wall integrity stresses agent (200 μg/ml and 500 μg/ml CR) at 37°C for 7 days. **(B)** Inhibition growth rate induced by oxidative stress agents of WT, Δ*set3-1*, Δ*set3-2,* and Δ*set3-com* strains. **(C)** Inhibition growth rate induced by cell wall integrity stress agent of WT, Δ*set3-1,* Δ*set3-2,* and Δ*set3-com* strains. Different letters represent *p* < 0.05.

To verify whether *A. flavus* Set3 was involved in stress responses, we measured several environmental stress responses by adding various stress agents into the tested media. As shown in Figures 6A,B, the Δ*set3-1* and Δ*set3-2* strains showed more endurance (*p* < 0.05) than WT and Δ*set3-com* strains when induced by oxidative stress agents (2.5 mM and 5 mM H_2_O_2_), suggesting that the Δ*set3-1* and Δ*set3-2* strains were less sensitive to the oxidative stress. Additionally, our results displayed that the relative growth inhibition of the deletion strains was significantly higher (*p* < 0.05) than that of WT and Δ*set3-com* strains when induced by cell wall integrity stress agent Congo Red (CR, 200 and 500 μg/ml) (Figures 6A,C). Whereas there was no inhibition growth difference among the WT, Δ*set3-1,* Δ*set3-2,* and Δ*set3-com* strains with the addition of osmotic stress (sodium chloride, NaCl) and genotoxic stress (methyl methanesulfonate, MMS) agents (*p* > 0.05) (data not shown). All these results suggested that Set3 participates in oxidative and cell wall stress responses in *A. flavus*.

### *Set3* Negatively Regulates Aflatoxin Production

**Figure 7:**
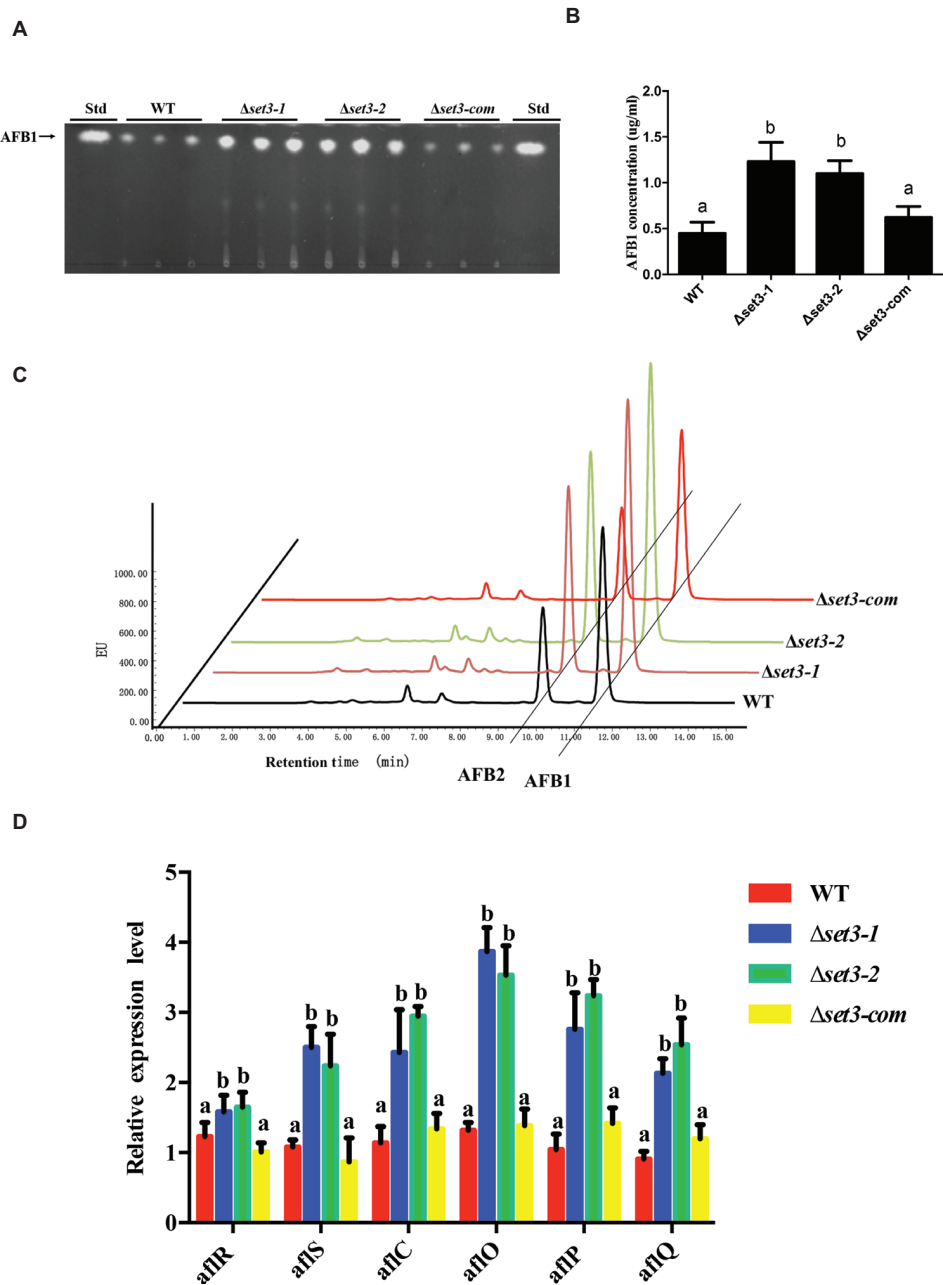
Aflatoxin production of WT, Δ*set3-1,* Δ*set3-2,* and Δ*set3-com* strains. **(A)** Aflatoxins were detected by thin-layer chromatography (TLC) after grown on YES media for 3 days at 28°C in the dark. **(B)** Relative aflatoxin production in (A) was qualified. **(C)** HPLC analysis of aflatoxin production in WT, Δ*set3-1,* Δ*set3-2,* and Δ*set3-com* strains after grown on YES media for 3 days at 28°C in the dark. **(D)** Transcript level of aflatoxin-related genes *aflR*, *aflS*, *aflC*, *aflO*, *aflP*, *aflQ* from WT, Δ*set3-1,* Δ*set3-2,* and Δ*set3-com* strains. Different letters represent *p* < 0.05.

To examine if Set3 plays a role in aflatoxin (AFs) production, content of AFs in Δ*set3-1* and Δ*set3-2* cultures as well as in WT and complemented strains were assayed. The results showed that deletion of *set3* gene resulted in a significant increase (>100%) (*p* < 0.05) in aflatoxin B1 (AFB1) levels in comparison with those in WT and Δ*set3-com* strains (Figures 7A,B). These findings were further confirmed by high performance layer chromatography (HPLC) analysis, showing both AFB1 and aflatoxin B2 (AFB2) production were upregulated in Δ*set3-1* and Δ*set3-2* strains (Figure 7C). In addition, we detected transcript levels of genes relevant to aflatoxin biosynthesis. The qRT-PCR results indicated that both Δ*set3-1* and Δ*set3-2* strains increased the transcript levels of the candidate genes for AFs biosynthesis, including *aflR*, *aflS*, *aflC*, *aflO*, *aflP,* and *aflQ*, when compared to that of WT and Δ*set3-com* strains (Figure 7D). These above results implied that *set3* negatively regulates AF production in *A. flavus*.

### *Set3* Is Involved in Crop Kernel Colonization

**Figure 8:**
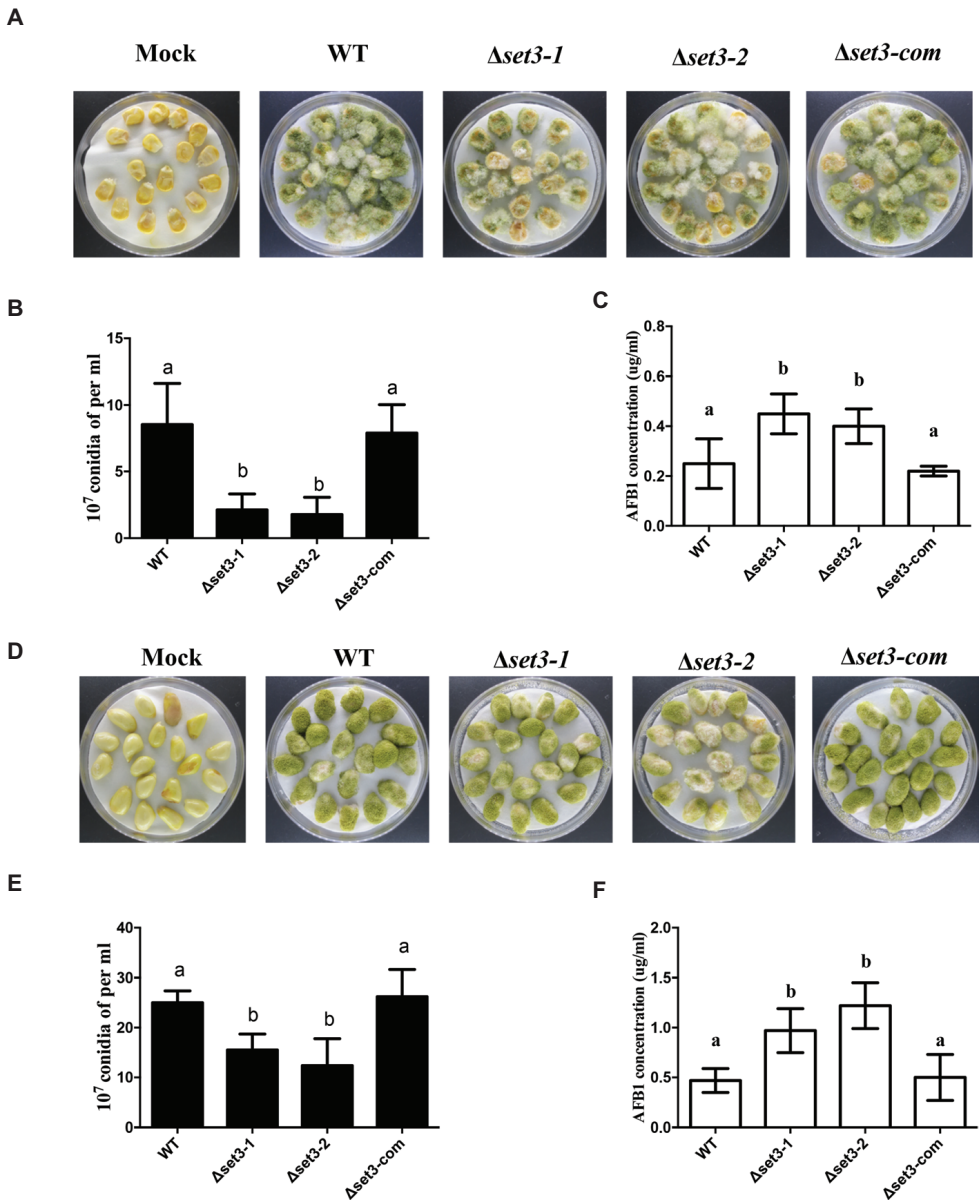
Crops infection of WT, Δ*set3-1,* Δ*set3-2,* and Δ*set3-com* strains. **(A)** Phenotype of all strains grown on living maize seeds after grown in darkness for 5 days. **(B)** Amount of conidia was measured from the infected maize seeds. **(C)** Aflatoxin production was detected from the infected maize seeds. **(D)** Phenotype of all strains grown on peanut seeds after 5 days in darkness. **(E)** Amount of conidia was measured from the infected peanut seeds. **(F)** Aflatoxin production was detected from the infected peanut seeds. Mock represents that crop kernels were inoculated with sterile water as a control group. Different letters represent *p* < 0.05.

To determine the roles of Set3 in kernel virulence, peanuts and maize kernel seeds were inoculated with WT strain, the Δ*set3-1,* Δ*set3-2,* and Δ*set3-com* strains. Visually, both the Δ*set3-1* and Δ*set3-2* strains showed less able to infect and sporulate on host seeds (Figures 8A,D). After 5 days inoculation at 28°C, we assayed conidia amount from the host seeds, and the results showed that Δ*set3-1* and Δ*set3-2* strains were impaired to generate the conidia in comparison with the WT and complemented strains (*p* < 0.05) (Figures 8B,E). The aflatoxin from the infected seeds was subsequently assessed, and the results in Figures 8C,F showed that the Δ*set3-1* and Δ*set3-2* strains produced more AF contents (*p* < 0.05) in both peanut and maize seeds. All these results indicated that *set3* in *A. flavus* is involved in colonization to crops.

## Discussion

During recent years, SET and PHD domain orthologs have been documented to play crucial roles in increasingly organisms from fungi to animals (Pijnappel et al., 2001; Hnisz et al., 2010; Nobile et al., 2014; Yun et al., 2014; McElroy et al., 2017). Our *in silico* analysis indicated that the predicted Set3 protein sequences were conserved within its corresponding homologs (Figure 1). *A. flavus* Set3 shows 100% identity to its homolog in important industrial fungus *Aspergillus oryzae*, and 61% identity to its homolog in the model *Aspergillus* species *A. nidulans*. Though it only shares 45% similarity with the model plant species *Arabidopsis thaliana* and 38% similarity with *Drosophila elegans*, the whole analyzed organisms harbor the conserved PHD and SET domain, implying that Set3 is important for survival. In yeast, Set3 is a non-essential gene, for survival, while with a mutant phenotype of defective transcription kinetics (Wang et al., 2002; Hnisz et al., 2012). Deletion of *upset* gene, the *Drosophila* homolog of SET3, was found to be lethal in both sexes in flies (McElroy et al., 2017). What’s more, MLL5 (SET3 homolog in mammals) has been linked to several different cellular processes, including cell cycle progression (Deng et al., 2007), hematopoiesis (Heuser et al., 2009), oncogenesis (Emerling et al., 2002), and DNA methylation (Yun et al., 2014). In this study, our results indicated that Set3 protein positively regulates conidiation, sclerotial development, and cell wall stress response, whereas it negatively controls AF biosynthesis and oxidative stress response in *A. flavus*.

Here, we investigated the effects of Set3 on the fungal biology in *A. flavus*. Deletion of *set3* gene produces more hyphal in *A. flavus* (Figure 3), which means that Set3 functions as a repressor for the hyphal development in filamentous fungi. These findings are consistent with the report on the pleiomorphic fungal pathogen, *Candida albicans*, which showed that the Set3/Hos2 histone deacetylase complex (Set3C) acts as a crucial repressor of the yeast-to-filament transition (Hnisz et al., 2010), and inactivation of *set3* gene resulted in biofilm perturbation in this fungus (Nobile et al., 2014). Our results also revealed that Set3 is a positive regulator of *A. flavus* asexual development, as a significant reduction in the conidial production of the Δ*set3* colonies was observed when compared to that of WT (Figure 4). These were accompanied by a reduction in expression of *brlA* and *abaA*, essential genes in the central regulatory pathway that controls asexual development (Adams et al., 1988, 1998). Unlike its positive roles in conidiation in *A. flavus*, Set3C represses genes in early/middle of the yeast sporulation program, including key meiotic regulators Ime2 and Ndt80 (Pijnappel et al., 2001). Besides conidiation, Set3 also engages in sclerotia formation (Figure 5A), sexual development structures that allow this fungus to survive extreme environmental conditions (Wicklow, 1987; Cary et al., 2012), and it was well supported by the obvious downregulation of the sclerotia-related transcription factors *nsdC* (Figure 5C) and *sclR* (Figure 5D). All these observations indicated that Set3 plays diverse roles in cellular functions of filamentous fungi.

In natural environments, cells can experience rapidly changing conditions and must correspondingly change their gene expression patterns to adapt (Feil and Fraga, 2012). Set3 binding was enriched for stress-related genes, and it plays both positive and negative roles in cell defense (Hnisz et al., 2012; Kim et al., 2012). Set3C was important in regulating gene induction during the stress response, including changes in the carbon sources (Kim et al., 2012), nitrogen starvation (Pijnappel et al., 2001), and DNA damage (Sharma et al., 2007). Here, deletion of *A. flavus set3* caused less sensitive to oxidative stress (Figure 6). Previous study showed that a paralog to Set3 known as Set4 also contains a PHD finger and a divergent SET domain, and it can interact with chromatin, which directly localizes to stress response genes upon regulating ROS (Tran et al., 2018). Oxidative stress response is highly related to reactive oxygen species (ROS) (Schieber and Chandel, 2014). Therefore, it is reasonable to infer that *A. flavus* Set3 regulates oxidative stress response in the same pathway. On the contrary, Δ*set3* mutants showed more sensitive to cell wall stress than WT (Figure 6). Exposure to Congo red (CR) lowers the content of cell wall chitin, and the effects of Set3 on *A. flavus* cell wall integrity may be due to its regulation of the cell wall chitin accumulation factor Smp1 or the oligosaccharyltransferase Stt3 (Hagiwara et al., 2011). All these results suggest the diverse roles of Set3 in environmental stress responses. Therefore, we postulate that Set3 is likely to contribute to each cell defense through distinct molecular mechanisms in *A. flavus*; however, further investigation will be required to reveal the mechanisms driving the stress-responsive regulation by Set3.

Although the biosynthesis pathway of AFs has been well characterized, the regulatory mechanism is complicated and has not been fully understood. Specially, the involvement of both SET and PHD domain protein was not reported yet in control of secondary metabolism. Our results found that inactivation of Set3 promoted AF production and its related genes’ expression (Figure 7), suggesting that Set3 acted as a repressor in AF biosynthesis. Set3 and HosA, as the core subunit of Set3C histone deacetylase complex, had been shown similar biological functions in most studies (Pijnappel et al., 2001; Cohen et al., 2008; Hnisz et al., 2010; Torres-Machorro et al., 2015). In another study, we identified a key Set3C histone deacetylase component HosA (homolog to Hos2) of *A. flavus*, unexpectedly, deletion of *hosA* seriously reduced the AF production. This might be due to that HosA was required for bounding directly to AF biosynthesis cluster genes (data unpublished). We speculated that Set3 and HosA were independently involved in regulation of AF biosynthesis, not only restricted to function as the Set3C complex, but also might play roles in other pathways or functional complexes to control AF biosynthesis. Functional data on SET domain proteins have related to chromatin regulation, and in certain cases, epigenetic mechanisms. Specifically, Set3 proteins have been identified as histone methyltransferase (Kim and Buratowski, 2009), and they participated in Hst1-Sum1 complex (Pijnappel et al., 2001). From the upregulation of AF biosynthesis regulatory genes *aflR* and *aflS* in Δ*set3* strains (Figure 7D), it is possible that inactivation of Set3 may cause alteration of regulatory genes for post-translation modification. Taking together, these results further revealed that regulatory mechanism for AFs biosynthesis is highly complicated.

Previous study had been shown that *C. albicans* Δ*set3* displayed strongly attenuated virulence in a mouse model of systemic infection (Hnisz et al., 2010), but the role of Set3 in virulence is still unknown in filamentous fungus. *A. flavus* has potential to infect oilseed crops by sporulation on injured seeds, therefore, to contaminate the hosts with AFs. Although the physiological significance of these SET domains remains unknown, Set3 may be relevant to fungal virulence of *A. flavus*, on the basis of the reduction of conidiation and increase of aflatoxin biosynthesis as a result of the inactivation of *set3*. This idea is further supported by the colonization phenotypes of the *set3* mutants on both peanut and maize seeds (Figure 8).

In conclusion, we identified a novel Set3 consisting of a functional SET and a PHD domain in *A. flavus*. Our results suggested that *A. flavus* Set3 plays important roles in reproduction, AFs biosynthesis, and fungal virulence and provides a novel sight for developing new fungal control strategies. Whereas further studies are required to discover the SET and PHD protein machinery and the molecular mechanism of Set3 cross-talk with the other crucial signal pathways in *A. flavus*.

## Author Contributions

HL, LW, and SW conceived and designed the experiment. HL, RS, KF, XL, YC, and LW performed the experiments. HL, LW, KY, and SW analyzed the data. HL, JT, FZ, KY, GY, and SW wrote the manuscript.

### Conflict of Interest Statement

XL was employed by the company Longyan City Corporation of Fujian Tobacco Corporation.

The remaining authors declare that the research was conducted in the absence of any commercial or financial relationships that could be construed as a potential conflict of interest.
